# Efficiency of DNA Isolation Methods Based on Silica Columns and Magnetic Separation Tested for the Detection of *Mycobacterium avium* Subsp. *Paratuberculosis* in Milk and Faeces

**DOI:** 10.3390/ma13225112

**Published:** 2020-11-12

**Authors:** Marketa Husakova, Petr Kralik, Vladimir Babak, Iva Slana

**Affiliations:** 1 Veterinary Research Institute, Hudcova 70, 621 00 Brno, Czech Republic; marketahusakova12@centrum.cz (M.H.); kralikp@vfu.cz (P.K.); babak@vri.cz (V.B.); 2 Department of Experimental Biology, Faculty of Science, Masaryk University, Kamenice 5, 625 00 Brno, Czech Republic; 3 Department of Hygiene and Technology of Food of Animal Origin and of Gastronomy, Faculty of Veterinary Hygiene and Ecology, University of Veterinary and Pharmaceutical Sciences, 612 42 Brno, Czech Republic

**Keywords:** *Mycobacterium avium* subsp. *paratuberculosis*, magnetic separation, silica columns, Johne’s disease, paratuberculosis, milk, faeces, DNA isolation, F57 PCR

## Abstract

Timely and reliable detection of animals shedding *Mycobacterium avium* subsp. *paratuberculosis* (MAP) should help to effectively identify infected animals and limit infection transmission at early stages to ensure effective control of paratuberculosis. The aim of the study was to compare DNA extraction methods and evaluate isolation efficiency using milk and faecal samples artificially contaminated by MAP with a focus on modern instrumental automatic DNA isolation procedures based on magnetic separation. In parallel, an automatic and manual version of magnetic separation and two methods of faecal samples preparation were compared. Commercially available DNA isolation kits were evaluated, and the selected kits were used in a trial of automatic magnetic beads-based isolation and compared with the manual version of each kit. Detection of the single copy element F*57* was performed by qPCR to quantify MAP and determine the isolation efficiency. The evaluated kits showed significant differences in DNA isolation efficiencies. The best results were observed with the silica column Blood and Tissue kit for milk and Zymo Research for faeces. The highest isolation efficiency for magnetic separation was achieved with MagMAX for both matrices. The magnetic separation and silica column isolation methods used in this study represent frequently used methods in mycobacterial diagnostics.

## 1. Introduction

Paratuberculosis (Johne’s disease), a chronic infectious intestinal disease caused by *Mycobacterium avium* subsp. *paratuberculosis* (MAP) occurs in dairy cattle and other ruminants worldwide and represents a major challenge for mycobacterial diagnostics. Clinical symptoms may develop after many years, making early diagnosis difficult [1,2]. Diagnosis of MAP infection is challenging because of the pathogen´s fastidious in vitro growth requirements and low-level intermittent shedding in faeces during the preclinical phase of the infection [3]. For example, a U.S. study found that 71% of cows were low shedders (<10 CFU/tube, i.e., <5 CFU/g), 10% were medium (10–50 CFU/tube), with 19% classified as high shedders (>50 CFU/tube) [4]. Detection of these “low- shedders” is important for effective control of paratuberculosis as these animals serve as sources of infection to susceptible calves [3]. Faeces are considered one of the most important samples for the diagnosis of paratuberculosis, because it is possible to identify subclinical and clinical animals via the shedding of MAP [5].

MAP in milk from an animal point of view represents a source of potential infection to calves, as animals are usually infected at a young age from contaminated milk or colostrum [6]. The current understanding of Johne’s disease transmission is that calves born to MAP-positive dams are at a higher risk of becoming infected; as such, dams are thought to excrete high quantities of MAP in colostrum and faeces, which may contaminate the calf during parturition or nursing [7]. However, recent findings [8] provide strong evidence that calves are at high risk for Johne’s disease even when dams are negative at the time of calving and seroconvert more than 12 months after a calf´s birth. MAP may also impact public human health, as the organism has been consistently found in people with Crohn´s disease, suggesting that this agent is zoonotic [9]. Milk is considered a potential transmission route to humans. Early investigations found that MAP was shed in low numbers (2–8 CFU/50 mL milk) in colostrum and milk from both clinically and subclinically infected animals [10,11,12]. However, commercial pasteurisation does not completely eliminate MAP from milk [13,14], nor does combined pasteurisation and desiccation in the preparation of infant formula [15]. Therefore, control must be implemented at a farm level to minimise exposure [16].

Polymerase chain reaction (PCR) has gained popularity for the diagnosis of paratuberculosis, with a sensitivity and specificity superior to culture. Moreover, culture is laborious and time-consuming [17,18]. However, a critical step in any direct PCR is the extraction method, with a matrix such as faeces or milk and an organism such as MAP making efficient extraction particularly challenging. The reasons for this include the presence of inhibitors in faeces or milk and the thick waxy MAP cell wall that makes extraction of DNA difficult. Inhibitors present in faeces include phytic acid, polysaccharides, or fat in milk that can lead to false-negative results by inhibiting amplification of DNA in PCR [19,20,21]. Another cause is the inadequate cell lysis of MAP, due to the characteristics of the MAP cell wall [22].

The use of a magnetic separation (MS) method especially in conjunction with PCR as a preferable detection method in routine diagnostics has risen in recent years. MS has become a high-throughput routine method in food and veterinary microbiology laboratories and is commonly used for the detection and isolation of pathogenic bacteria [23,24,25,26]. This method involves a reversible interaction between target cells and magnetic particles. These complexes are easy to separate from sample by the application of a strong magnetic field. The selectivity of capture is assessed by determining the efficiency of capture and depends on the bead characteristics (composition, size, concentration, and surface modification) or the nature of the coating ligand (polyclonal ormonoclonal antibody, biotinylated, or nonbiotinylated peptide) [26].

The silica column approach is based on a membrane that utilizes the binding properties of a silica-based membrane. DNA adsorbs to the membrane in the presence of high concentrations of chaotropic salt, which remove water from hydrated molecules in solution [27].

The aims of this study were to conduct a comparison of DNA isolation efficiency obtained using commercially available DNA isolation kits based on two different approaches—magnetic separation and silica columns: (1) in spiked milk and faeces, (2) in faecal samples and pellets from faecal suspensions, (3) with a comparison of manual and automatic magnetic separation, and (4) to evaluate a comparison of DNA concentration and purity of model contamination by MAP.

## 2. Materials and Methods

### 2.1. Preparation of MAP Culture for Artificial Contamination Experiments

For the artificial contamination of milk and faecal samples (both types of samples freshly collected), MAP strain 7072 originating from deer pulmonary lymph nodes of clinically infected animals from the strain collection of the OIE was used.

Reference Laboratory for Paratuberculosis (Veterinary Research Institute, Brno, Czech Republic) was used. The culture was grown on Herrold’s egg yolk medium (HEYM) with 2 µg/mL of Mycobactin J and incubated at 37 °C for 6 weeks. Subsequently, the grown MAP culture was resuspended in 1 mL of Tris-EDTA (TE) buffer and homogenised using twelve 1 mm zirconia-silica beads in a MagNA Lyser instrument (Roche Diagnostics GmbH, Mannheim, Germany) at 6400 rpm for 10 s. The MAP suspension was centrifuged at 100× *g* for 30 s to remove big clumps and the upper part of the liquid was taken. The optical density of the collected suspension was determined on a BioPhotometer (Eppendorf) at 600 nm and the MAP cell concentration was estimated according to the formula OD600 = 0.1 corresponding to 10^8^ MAP cells/mL. Subsequently, the suspension was diluted [28] to obtain a concentration range of about 10^7^–10^4^ MAP cells/mL. Prepared serial dilutions of MAP suspensions were used for artificial contamination. To assess the real quantity of MAP in dilutions, 300 µL of each was homogenized using 350 mg of 0.1 zirconia-silica beads and a MagNA Lyser instrument (Roche Diagnostics GmbH, Mannheim, Germany) at 6400 rpm for 1 min and centrifuged at 12,000× *g* for 5 min. The supernatant served as a template to F57 qPCR [29].

### 2.2. Preparation of Milk and Faecal Samples Used for Artificial Contamination

Milk pellets were prepared from whole milk (sample volume 50 mL), as previously described by Slana et al. [29]. Artificial contamination was performed using 10 µL of a MAP cell suspension of the concentration range of 10^7^–10^5^ MAP cells/mL per milk pellet. Each concentration was carried out in triplicate. Faecal samples originating from a beef cattle farm with no history of paratuberculosis presence were used for the preparation of either faecal samples or faecal pellets. Faeces were processed directly, and 200 mg was utilised in analyses. Pellets were prepared from 5 g of a faecal sample, which was resuspended in 30 mL of distilled water with vortexing. After 10 min of sedimentation, 1.5 mL of the supernatant was transferred into new 2 mL microcentrifuge tubes. Consequently, the samples were centrifuged at 11,300× *g* for 10 min. The supernatant was poured off and the remaining pellets were used for artificial contamination experiments [28], using 10 µL of a MAP cell suspension in a concentration range of 10^6^ to 10^4^ MAP cells/mL per faecal pellet or faecal sample. Each concentration used for artificial contamination was analysed in biological duplicate.

### 2.3. DNA Isolation and qPCR Detection of MAP

Isolation of total DNA from milk and faeces was performed according to manufacturers’ protocols. Individual kits were selected based on manufacturers’ recommendations for the relevant biological material or according to respective publications [30,31,32]. Because of a thick, waxy mycobacterial cell wall, a homogenisation step with 0.1 mm zirconia-silica beads in a MagNA Lyser instrument (Roche Diagnostics GmbH, Mannheim, Germany) was added to isolation protocols. Isolation of DNA from milk and faecal samples and pellets was performed manually using 7 (5 MS kits, 2 silica membrane columns kits; Supplementary Information Table S1) and 12 (4 MS kits, 8 silica membrane columns kits; Supplementary Information Table S2) commercially available kits, respectively. Automatic isolation of DNA from milk and faecal pellets was performed using 5 commercially available MS kits (Supplementary Information Table S3) and an IDEAL 32 extraction robot (ID Vet Genetics, Grabels, France).

Isolated DNA was analysed by home-made developed qPCR targeting the MAP specific single copy element F57. All tested samples were tested in technical duplicates and quantified as described by Slana et al. [29]. The DNA isolation efficiency for each kit was calculated as the quotient of the acquired quantity of MAP cells after DNA isolation and the theoretical number of MAP cells spiked to each sample. The concentration and purity of isolated DNA were measured using the spectrophotometer NANODROP 2000c (Thermo Fisher Scientific, Vilnius, Lithuania). All acquired data were statistically analysed using the program STATISTICA 13.2 (Dell, Inc., Tulsa, Oklahoma USA) and Friedman, Dunn post-hoc, and Scheirer–Ray–Hare tests. *p*-values lower than 0.05 were considered statistically significant.

## 3. Results

### 3.1. Efficiency of Manual MAP DNA Isolation

For determination of DNA isolation efficiency, three high concentrations of MAP cells were used. Mean and median values for each MAP cell concentration were calculated from the data of the F57 qPCR assay (Supplementary Information Tables S4, S5, S6). In general, with decreasing MAP cell concentration, the efficiency of DNA isolation decreased proportionally with all commercial kits used (Supplementary Information Tables S7 and S8).

**Figure 1:**
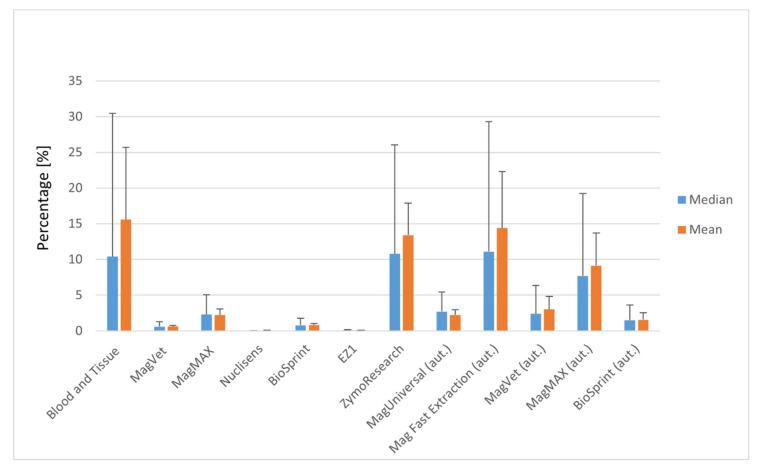
Efficiency of *Mycobacterium avium* subsp. *paratuberculosis* (MAP) DNA isolation in milk using selected isolation kits: DNeasy^®^ Blood and Tissue kit (QIAGEN, Hilden, Germany), MagVet Mycobacterium paratuberculosis Isolation Kit (LSI, Lissieu, France), MagMAX™ Total Nucleic Acid Isolation Kit (Applied Biosystems by Thermo Fisher Scientific, Vilnius, Lithuania), Nuclisens Magnetic Extraction Reagents (Biomérieux, Marcy-l’Étoile, France), BioSprint 96 One-For-All-Vet (QIAGEN, Hilden, Germany), EZ1 DNA Tissue Kit (QIAGEN, Hilden, Germany), ZR Quick-DNA Fecal Soil Microbe Microprep Kit (Zymo Research, Tustin, CA, USA), ID Gene™ Mag Universal Extraction Kit (ID vet Genetics, Grabels, France), and ID Gene™ Mag Fast Extraction Kit (ID vet Genetics, Grabels, France). aut.—automatically processed. Error bars represent the 90th percentile and standard deviation for median and mean, respectively.

For manual silica column-based DNA isolation from milk, the Blood and Tissue silica column isolation kit was the most effective. The mean value for this kit was 15.60%. A reasonable isolation efficiency was also recorded for the silica column Zymo Research kit (Tustin, California, USA; 13.40%), which had a similar efficiency (tens of percent). A mean efficiency about one order of magnitude lower (units of percent) was acquired with the MagMAX isolation kit (Applied Biosystems by Thermo Fisher Scientific, Vilnius, Lithuania; 2.20%). The remaining kits used for isolation of MAP DNA from milk MagVet (LSI Lissieu, France), Nuclisens (Biomérieux Marcy-l’Étoile, France), BioSprint (QIAGEN, Hilden, Germany), EZ1 (QIAGEN, Hilden, Germany) achieved much lower mean isolation efficiencies ranging from 0.03% (Nuclisens) to 0.83% (BioSprint) and were found to be inappropriate for this type of pathogen and matrix. Of isolation kits based on MS, the MagMAX was the best with an isolation efficiency when considering level of units of percent (Figure 1).

**Figure 2:**
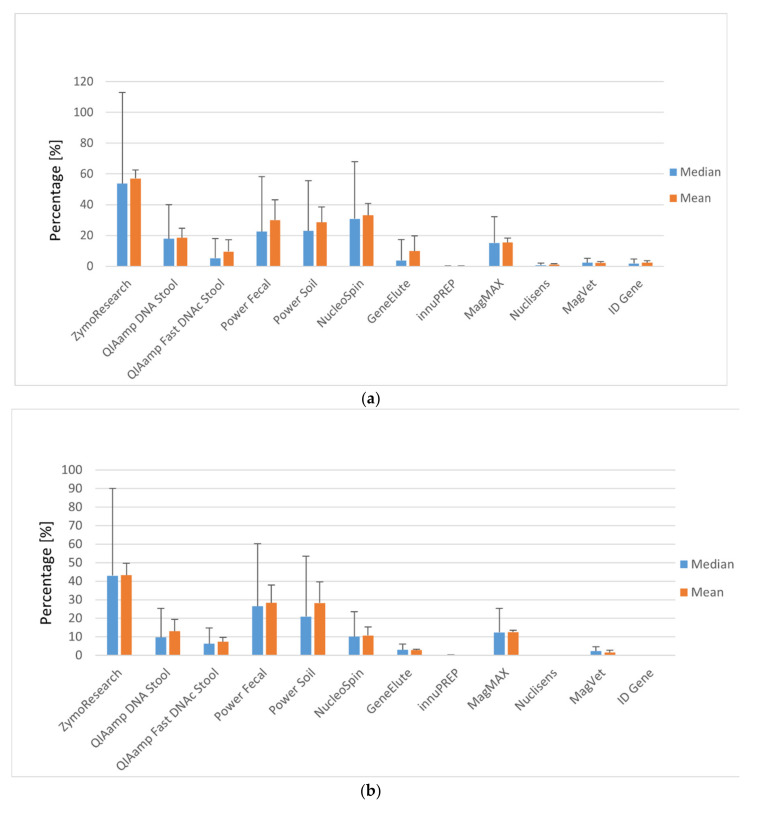
(**a**) Efficiency of MAP DNA isolation in faecal pellets using selected isolation kits: ZR Quick-DNA Fecal Soil Microbe Microprep Kit (Zymo Research, Tustin, CA, USA), QIAamp DNA Stool Mini Kit (QIAGEN, Hilden, Germany), QIAamp Fast DNA Stool Mini Kit (QIAGEN, Hilden, Germany), Power Fecal DNA Kit (QIAGEN, Hilden, Germany), DNeasy Power Soil Kit (QIAGEN, Hilden, Germany), NucleoSpin DNA Stool (Macherey-Nagel, Düren, Germany), Gen Elute Stool DNA Isolation Kit (Sigma-Aldrich, St. Louis, MO, USA), Innu PREP Stool DNA kit (Analytik Jena, Berlin, Germany), MagMAX™ Total Nucleic Acid Isolation Kit (Applied Biosystems by Thermo Fisher Scientific, Vilnius, Lithuania), Nuclisens Magnetic Extraction Reagents (Biomérieux, Marcy-l’Étoile, France), MagVet Mycobacterium paratuberculosis Isolation Kit (LSI, Lissieu, France), and ID Gene Mag Paratuberculosis Extraction Kit (ID vet Genetics, Grabels, France). Error bars represent the 90th percentile and standard deviation for median and mean, respectively. (**b**) Efficiency of MAP DNA isolation in faecal samples using selected isolation kits; for detailed isolation kit information, see (**a**). Error bars represent the 90th percentile and standard deviation for median and mean, respectively.

Generally, DNA isolated from faecal pellets was found to provide higher isolation efficiencies than DNA isolated directly from faeces for all kits tested (Figure 2). Half of the tested isolation kits achieved efficiencies in the order of tens of percent, and three kits had efficiencies in the order of units of percent. Three isolation kits (Nuclisens, innuPREP, ID Gene) were unsuitable for DNA extraction from faecal samples (showed no detection of targeted DNA or inhibition). The best mean results gained using faecal pellets were achieved with the silica column Zymo Research isolation kit with a yield of 57.07%. Very favourable values were also obtained with the kits NucleoSpin (33.13%), Power Fecal (29.93%), and Power Soil (28.53%), all using the silica column method. A reasonable mean efficiency was also recorded for the silica column kit QIAamp DNA Stool (18.50%) and lower values for QIAamp Fast DNA Stool (9.53%) and GeneElute (9.87%), whereas the best magnetic separation method result was achieved with MagMAX (15.50%; Supplementary Information Table S5). In comparison, the results of faecal samples with silica column isolation achieved generally lower efficiencies in the Zymo Research (43.27%), NucleoSpin (10.73%), or QIAamp DNA Stool kits (13.00%), except for Power Fecal (28.33%) and Power Soil (28.33%) kits, which achieved similar mean efficiencies as for faecal pellets. Regarding the magnetic separation method, the best mean result was acquired again using MagMAX (12.57%; Supplementary Information Table S6).

### 3.2. Manual versus Automatic Magnetic Separation of MAP DNA

**Figure 3:**
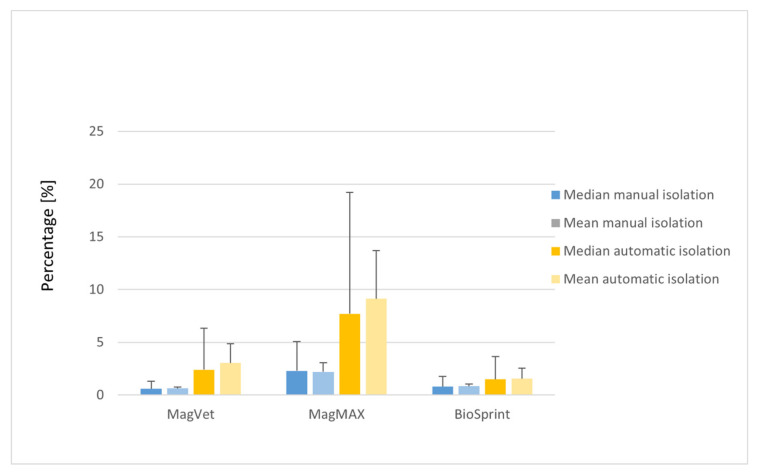
Comparison of MAP DNA isolation efficiency of manual and automatic magnetic separation in milk using selected isolation kits; for detailed isolation kit information, see Figure 1. Error bars represent the 90th percentile and standard deviation for median and mean, respectively.

Three identical isolation kits with manual isolation (MagVet, MagMAX and BioSprint) and two others (Mag universal and Mag Fast Extraction kits) were tested on milk using an automatic isolation robot (Supplementary Information Table S9). In the automated isolation, the Mag Fast Extraction kit and MagMAX isolation kit had the highest mean isolation efficiency (14.40 and 9.13%, respectively; Figure 1, Supplementary Information Table S10). The remaining isolation kits tested with the automatic robot had a mean efficiency in units of percent. To compare manual and automatic MS of MAP DNA in milk and faeces, three kits were used for both, including MagVet, MagMAX, and BioSprint (Supplementary Information Table S11). In the case of milk, higher mean values of DNA isolation efficiencies were reached using automatic MS than by manual MS (Figure 3, Supplementary Information Table S12). The best result was obtained with the MagMAX kit (9.13%) during automatic MS of MAP DNA from milk.

Regarding automatic MS of MAP DNA in faeces, only faecal pellets were used because manufacturers of the kits do not recommend using crude faeces because of the high risk of inhibitors to subsequent qPCR analysis. This was proven experimentally in this study. Inhibition of the qPCR reaction was also observed for faecal pellets in all kits used except for the MagMAX kit, but the percentage of isolation efficiency reached was below 1.0% (data not shown). Thus, in this case, manual MS is considered to be a better approach than automatic MS for isolation of MAP DNA from faeces.

The concentration and purity (A260/280 absorbance ratio) of isolated DNA from milk and faecal samples were measured and the results are summarised in the Supplementary Information (Tables S13–S15, Figures S1 and S2).

## 4. Discussion

This study describes a comparison of isolation efficiencies of MAP DNA from milk and faeces using the two most common isolation approaches for this purpose—the silica column isolation method and magnetic separation. These methods were used in combination with real-time qPCR in order to evaluation the best methodology used for MAP detection in samples with the most frequent occurrence of the pathogen. Our findings proved the limits of magnetic separation technology and revealed silica column ZR Quick-DNA Fecal Soil Microbe Microprep Kit from Zymo Research to be the best option for MAP diagnostics. Inhibitory sample components such as fat or Ca^2+^ ions in milk make isolation of mycobacteria from this matrix difficult [33]. Moreover, isolation procedures are often designed for the milk matrix itself and not for mycobacteria diluted in milk. This creates a need to concentrate mycobacteria from a high volume of milk into the small volume of a milk pellet. The silica column Blood and Tissue kit had the highest mean isolation efficiency (15.60%) from milk followed by the automatic MS Mag Fast Extraction Kit (14.40%) and silica column Zymo Research kit (13.40%). Interestingly, the MagMAX kit achieved higher efficiency when used in automatic MS mode (9.13%) than in manual (2.20%).

The majority of the kits used for isolation of MAP DNA from milk acquired very low mean percentage values for isolation efficiency. This could suggest general impropriety of some types of kits for this type of matrix. For example, Foddai et al. [24] highlights that not all magnetic separation approaches developed for MAP perform equally well. Capture is assessed by determining its efficiency and depends on bead characteristics (composition, size, concentration, or surface modification).

In contrast, the use of automatic MS for faecal samples or pellets appeared to be inappropriate, indicating a larger effect of faecal inhibitors than those of milk. Regarding automatic MS of MAP DNA from faeces, we encountered the problem of insufficient removal of inhibitors in washing steps. This was documented by an observation of turbidity in the elution buffer step, while the washing buffer steps remained clear. Thus, the problem could either be in the cleaning ability of washing buffers or adhesion of the magnetic beads, which bind not only to DNA, but also inhibitory sample components. This issue was more pronounced in manually processed isolation when a lower isolation efficiency was recorded in comparison with an automatic setup. Curiously, the best kit used for faeces, the silica column Zymo Research, which achieved very high mean isolation efficiencies in both faecal pellets (57.07%) and faecal samples (43.27%), acquired only 13.40% in the case of milk. Leite et al. [5] also describes the Zymo Research kit as the best of the six commercial kits tested for isolation of MAP DNA from faecal samples. On the other hand, the highest mean isolation efficiency for MAP DNA in faeces using the magnetic separation method was achieved with the manually performed MagMAX isolation kit (15.50%). Similar results were obtained by Okwumabua et al. [30], who consider the MagMAX procedure as technically simple and not time-consuming with highly reproducible results. The study also recommended the kit directly for isolation of MAP DNA, which our laboratory also confirms. Another study, Chui et al. [34], comparing four extraction methods for the identification of MAP DNA in faecal samples, also found MS and silica column isolation to be among the best two methods for this purpose.

It must be noted that the majority of DNA isolation kits are not designed for isolation of bacterial DNA from faeces, but for total DNA from faeces. Moreover, bacterial DNA represents only a small fraction of the total DNA in faeces. For illustration, the MAP genome is 4.8 Mbp long, while the bovine (*Bos taurus*) genome is 2.87 Gbp long [35]. Thus, the MAP genome represents a minor part (approximately 591 times less) of a sample compared with animal and plant genomes (that may be present in faeces). Therefore, the superior performance of the Zymo Research kit may be because of the fact that it is primarily intended for pathogen DNA isolation compared with the rest of the kits. In order to test suitable sample input, in addition to faecal pellets, raw faecal samples were also used with 200 mg of faeces per sample. However, this approach appeared to be less appropriate, as the use of faecal pellets exhibited higher isolation efficiencies. On the other hand, some results obtained with faecal samples displayed inhibition of qPCR reaction owing to the high concentration of inhibitory sample components present in faeces. Moreover, the presence of mycobacteria is not homogenous throughout crude faecal samples, thus input of 200 mg of faeces cannot be considered a fully representative sample. Consequently, the preparation of faecal pellets originating from a higher amount of faeces (5 g) was chosen as a preferable method. To make samples comparable, it was also necessary to directly artificially contaminate the final faecal pellets and not the suspension.

Data from a similar study carried out by Sting et al. [36] suggest the success of detection of MAP in faeces does not depend on the amount of faeces used, but instead on the concentration of MAP and the elimination of PCR inhibitors. The study of Leite et al. [5] reflects the analogous thought that sample size does not seem to be a determining factor for the sensitivity of different extraction kits. In addition, Sting et al. [36] suggest that, for the same purpose, generating bacterial pellets might also be adequate and might reveal opportunities for removing PCR inhibitors, which corresponds with our observations. Our results also correspond with the observations of Leite et al. [5] regarding the necessity of a physical disruption (bead beating) step for mycobacteria, as without this step, detecting MAP DNA is very difficult.

In general, the mean isolation efficiency of the silica column isolation method was far higher than that of magnetic separation, especially for faeces acquiring from 57.07% compared with 15.50%. However, the difference between the best mean result of silica column isolation and magnetic separation was not so dramatic in milk, with values of 15.60% compared with 14.40%. Regarding the magnetic separation method, the automatic MS Mag Fast Extraction Kit was the most efficient. The highest mean isolation efficiency for manual MS was achieved with the MagMAX kit. Besides this, our study suggests the suitability of the use of manual or automatic magnetic separation strongly depends on the type of matrix. For example, automatic magnetic separation of MAP DNA from faeces is still complicated by inhibitors and requires a focus on improvements in this method. Overall, the best solation kit of this study was considered to be the silica column Zymo Research kit, which is multi-purpose and suitable for a broad range of matrixes, milk and faeces included, and was subsequently selected as the kit for routine analysis in our laboratory.

DNA concentration and purity are frequently used indicators of DNA isolation efficiency from various matrices. This is true for DNA isolated for, e.g., genomic studies, where the exact amount of DNA is required as the input to different analyses. However, in the area of detection and particularly pathogen detection, there is no correlation between the yield and purity of isolated DNA and its amplifiability in downstream applications like qPCR. In other words, pure and highly concentrated DNA does not automatically mean good performance in qPCR. The results showed that there is no correlation between DNA yield and purity and the results from qPCR. Moreover, highly concentrated and pure DNA samples showed poor qPCR amplification. This specific issue must be considered when working with complex, difficult, and heterogeneous matrices like milk and faeces as there are many compounds in these matrices that can inhibit qPCR even in trace amounts. Inhibitors to the PCR reaction in faeces such as complex polysaccharides, bilirubin, or humic acids can influence measurements because they also absorb light at 260 nm and participate in qPCR inhibition [19,20,21].

## 5. Conclusions

Detection and quantification of MAP in faeces and milk by qPCR is a key component in the new generation of control programmes for paratuberculosis. Not only in terms of sensitivity for the detection of MAP in pooled faecal samples, but also in precise quantification of MAP in individual faeces for the correct identification of truly infected and “contaminated” animals. In parallel, the presence of MAP in milk is a very important health issue, concerning all members in the milk production chain, starting with milk producers and processors and ending with consumers.

A key component of DNA-based methods, which defines its sensitivity, is DNA isolation. It can be concluded that the ZR Quick-DNA Fecal Soil Microbe Microprep Kit from Zymo Research represents a universal tool for the detection of MAP in both matrices at a small to medium scale. It is robust, user-friendly, and primarily intended for bacterial DNA isolation.

DNA isolation kits based on magnetic separation, regardless of manual or automatic versions, were shown to be quite average, even second-rate in their performance. These kits in an automated regime can provide desired high-throughput DNA isolation. However, the efficiency to extract bacterial DNA from matrices such as faeces or milk could be a bottleneck in these approaches. Despite this fact, increasingly more companies provide an automatic isolation approach suitable for a broad range of sample matrices and pathogens, mycobacteria included. Our results suggest that the magnetic separation approach remains problematic for the isolation of MAP DNA, possibly because of high heterogeneity of faeces (it is difficult to define an average faecal sample), which is the consequence of feed composition and presence of potent inhibitors. It is expected that these technologies are likely to undergo significant development in the future and, therefore, automated magnetic separation technologies for faeces and milk may become preferable in the future.

## Supplementary Materials

The following are available online at https://www.mdpi.com/1996-1944/13/22/5112/s1. Figure S1: Distribution of DNA purity values of selected isolation kits for MAP DNA isolation from milk; Figure S2: Distribution of DNA purity values of selected isolation kits for MAP DNA isolation from faeces; Table S1: List of isolation kits used for isolation of MAP DNA from milk; Table S2: List of isolation kits used for isolation of MAP DNA from faeces; Table S3: List of isolation kits used for automatic magnetic separation of MAP DNA from milk and faeces; Table S4: Data derived from isolation efficiency of MAP DNA from milk, unit:percentage [%]; Table S5: Data derived from isolation efficiency of MAP DNA from faecal pellets, unit:percentage [%]; Table S6: Data derived from isolation efficiency of MAP DNA from faecal samples, unit: percentage [%]; Table S7: Efficiency of MAP DNA isolation from milk; Table S8: Comparison of efficiency of MAP DNA isolation from faeces; Table S9: Automatic magnetic separation of MAP DNA in milk; Table S10: Data derived from isolation efficiency of MAP DNA automatic magnetic separation from milk, unit: percentage [%]; Table S11: Comparison of manual and automatic magnetic separation of MAP DNA in milk; Table S12: Comparison of isolation efficiency derived data of MAP DNA manual and automatic magnetic separation from milk, unit:percentage [%]; Table S13: Concentration and purity of nucleic acids isolated from milk; Table S14: Concentration and purity of nucleic acid isolated from faecal pellets; Table S15: Concentration and purity of nucleic acid automatically isolated from milk.
