# Supplementary material for: Efficiency of DNA Isolation Methods Based on Silica Columns and Magnetic Separation Tested for the Detection of Mycobacterium avium Subsp. Paratuberculosis in Milk and Faeces
Source: Materials (Basel). 2020 Nov 12;13(22):5112. doi: 10.3390/ma13225112 (PMC7697941; doi:10.3390/ma13225112)
Supplement: Supplementary file 1 [file materials-13-05112-s001.pdf]

materials

Supplementary Materials

Efficiency of DNA Isolation Methods Based on Silica Columns and Magnetic Separation Tested for the Detection of *Mycobacterium Avium* Subsp. *Paratuberculosis* in Milk and Faeces

Marketa Husakova [1,2], Petr Kralik [1,3] Vladimir Babak [1], and Iva Slana [1,*]

[1] Veterinary Research Institute, Hudcova 70, 621 00 Brno, Czech Republic; marketahusakova12@centrum.cz (M.H.); kralikp@vfu.cz (P.K.); babak@vri.cz (V.B.)
[2] Department of Experimental Biology, Faculty of Science, Masaryk University, Kamenice 5, 625 00 Brno, Czech Republic
[3] Department of Hygiene and Technology of Food of Animal Origin and of Gastronomy, Faculty of Veterinary Hygiene and Ecology, University of Veterinary and Pharmaceutical Sciences, 612 42 Brno, Czech Republic
* Correspondence: slana@vri.cz; Tel.: +420777786711

Table S1. List of isolation kits used for isolation of MAP DNA from milk.

Kit Number	Name of the Kit (company)	Isolation Method
1	DNeasy® Blood and tissue kit (QIAGEN, Hilden, Germany)	silica columns
2	ZR Quick-DNA Fecal Soil Microbe Microprep Kit (Zymo Research, Tustin, CA, USA)	silica columns
3	MagVet *Mycobacterium paratuberculosis* Isolation Kit (LSI, Lissieu, France)	magnetic separation
4	MagMAX™ Total Nucleic Acid Isolation Kit (Applied Biosystems by Thermo Fisher Scientific, Vilnius, Lithuania)	magnetic separation
5	Nuclisens Magnetic Extraction Reagents (Biomérieux, Marcy-l'Étoile, France)	magnetic separation
6	BioSprint 96 One-For-All-Vet (QIAGEN, Hilden, Germany)	magnetic separation
7	EZ1 DNA Tissue Kit (QIAGEN, Hilden, Germany)	magnetic separation

Table S2. List of isolation kits used for isolation of MAP DNA from faeces.

Kit Number	Name of the Kit (company)	Isolation Method
1	ZR Quick-DNA Fecal Soil Microbe Microprep Kit (Zymo Research, Tustin, CA, USA)	silica columns
2	QIAamp DNA Stool Mini Kit (QIAGEN, Hilden, Germany)	silica columns
3	QIAamp Fast DNA Stool Mini Kit (QIAGEN, Hilden, Germany)	silica columns
4	Power Fecal DNA Kit (QIAGEN, Hilden, Germany)	silica columns
5	DNeasy Power Soil Kit (QIAGEN, Hilden, Germany)	silica columns
6	NucleoSpin DNA Stool (Macherey-Nagel, Düren, Germany)	silica columns

7	Gen Elute Stool DNA Isolation Kit (Sigma-Aldrich, St. Louis, MO, USA)	silica columns
8	Innu PREP Stool DNA kit (Analytik Jena, Berlin, Germany)	silica columns
9	MagMAX Total Nucleic Acid Isolation Kit (Applied Biosystems by Thermo Fisher Scientific, Vilnius, Lithuania)	magnetic separation
10	Nuclisens Magnetic Extraction Reagents (Biomérieux, Marcy-l'Étoile, France)	magnetic separation
11	MagVet Mycobacterium paratuberculosis Isolation Kit (LSI, Lissieu, France)	magnetic separation
12	ID Gene Mag Paratuberculosis Extraction Kit (ID vet Genetics, Grabels, France)	magnetic separation

Table S3. List of isolation kits used for automatic magnetic separation of MAP DNA from milk and faeces.

Kit Number	Name of the Kit (company)
1	ID Gene™ Mag Universal Extraction Kit (ID vet Genetics, Grabels, France)
2	ID Gene™ Mag Fast Extraction Kit (ID vet Genetics, Grabels, France)
3	MagVet *Mycobacterium paratuberculosis* Isolation Kit (LSI, Lissieu, France).
4	MagMAX™ Total Nucleic Acid Isolation Kit (Applied Biosystems by Thermo Fisher Scientific, Vilnius, Lithuania).
5	BioSprint 96 One-For-All-Vet (QIAGEN, Hilden, Germany).

Table S4. Data derived from isolation efficiency of MAP DNA from milk, unit:percentage [%].

Kit	Isolation Efficiency	Median	Mean	Upper Quartile	Lower Quartile	Standard Deviation
Blood and Tissue[a]	29.7<br>10.4<br>6.7	10.4	15.60	20.05	8.55	10.08
MagVet[b]	0.8<br>0.6<br>0.5	0.6	0.63	0.7	0.55	0.12
MagMAX[c]	3.2<br>2.3<br>1.1	2.3	2.20	2.75	1.7	0.86
Nuclisens[d]	0.1<br>0<br>0	0	0.03	0.05	0	0.05
BioSprint[e]	1.1<br>0.8<br>0.6	0.8	0.83	0.95	0.7	0.21
EZ1[f]	0.1<br>0.1<br>0.1	0.1	0.10	0.1	0.1	0.00
ZymoResearch[g]	19.7<br>9.7<br>10.8	10.8	13.40	15.25	10.25	4.48

[a]DNeasy® Blood and tissue kit (QIAGEN, Hilden, Germany); [b]MagVet *Mycobacterium paratuberculosis* Isolation Kit (LSI, Lissieu, France); [c]MagMAX™ Total Nucleic Acid Isolation Kit (Applied Biosystems by Thermo Fisher Scientific, Vilnius, Lithuania); [d]Nuclisens Magnetic Extraction Reagents (Biomérieux, Marcy-l'Étoile, France); [e]BioSprint 96 One-For-All-Vet (QIAGEN, Hilden, Germany); [f]EZ1 DNA Tissue Kit (QIAGEN, Hilden, Germany); [g]ZR Quick-DNA Fecal Soil Microbe Microprep Kit (Zymo Research, Tustin, CA, USA).

Table S5. Data derived from isolation efficiency of MAP DNA from faecal pellets, unit:percentage [%].

Kit	Isolation Efficiency	Median	Mean	Upper Quartile	Lower Quartile	Standard Deviation
ZymoResearch[a]	64.8<br>52.7<br>53.7	53.7	57.07	59.25	53.2	5.48
QIAamp DNA Stool[b]	26.4<br>11.3<br>17.8	17.8	18.50	22.1	14.55	6.18
QIAamp Fast DNA Stool[c]	20.1<br>3.2<br>5.3	5.3	9.53	12.7	4.25	7.52
Power Fecal[d]	48.5<br>22.6<br>18.7	22.6	29.93	35.55	20.65	13.22
Power Soil[e]	42.4<br>23<br>20.2	23	28.53	32.7	21.6	9.87
NucleoSpin[f]	43.5<br>30.8<br>25.1	30.8	33.13	37.15	27.95	7.69
GeneElute[g]	23.7<br>3.7<br>2.2	3.7	9.87	13.7	2.95	9.80
innuPREP[h]	0<br>0.1<br>0.1	0.1	0.07	0.1	0.05	0.05
MagMAX[i]	19<br>12.4<br>15.1	15.1	15.50	17.05	13.75	2.71
Nuclisens[j]	2<br>0.7<br>0.7	0.7	1.13	1.35	0.7	0.61
MagVet[k]	3.1<br>1.2<br>2.4	2.4	2.23	2.75	1.8	0.78
ID Gene[l]	4<br>1.8<br>1.1	1.8	2.30	2.9	1.45	1.24

[a]ZR Quick-DNA Fecal Soil Microbe Microprep Kit (Zymo Research, Tustin, CA, USA); [b]QIAamp DNA Stool Mini Kit (QIAGEN, Hilden, Germany); [c]QIAamp Fast DNA Stool Mini Kit (QIAGEN, Hilden, Germany); [d]Power Fecal DNA Kit (QIAGEN, Hilden, Germany); [e]DNeasy Power Soil Kit (QIAGEN, Hilden, Germany); [f]NucleoSpin DNA Stool (Macherey-Nagel, Düren, Germany); [g]Gen Elute Stool DNA Isolation Kit (Sigma-Aldrich, St. Louis, MO, USA); [h]Innu PREP Stool DNA kit (Analytik Jena, Berlin, Germany); [i]MagMAX™ Total Nucleic Acid Isolation Kit (Applied Biosystems by Thermo Fisher Scientific, Vilnius, Lithuania); [j]Nuclisens Magnetic Extraction Reagents (Biomérieux, Marcy-l'Étoile, France); [k]MagVet *Mycobacterium paratuberculosis* Isolation Kit (LSI, Lissieu, France); [l]ID Gene Mag Paratuberculosis Extraction Kit (ID vet Genetics, Grabels, France).

Table S6. Data derived from isolation efficiency of MAP DNA from faecal samples, unit: percentage [%].

Kit*	Isolation Efficiency	Median	Mean	Upper Quartile	Lower Quartile	Standard Deviation
ZymoResearch	51.2<br>43<br>35.6	43	43.27	47.1	39.3	6.37
QIAamp DNA Stool	21.8<br>9.7<br>7.5	9.7	13.00	15.75	8.6	6.29
QIAamp Fast DNA Stool	6.3<br>5.2<br>10.5	6.3	7.33	8.4	5.75	2.28
Power Fecal	41<br>26.5<br>17.5	26.5	28.33	33.75	22	9.68
Power Soil	44.2<br>19.6<br>20.9	20.9	28.33	32.55	20.25	11.30
NucleoSpin	16.7<br>10.1<br>5.4	10.1	10.73	13.4	7.75	4.63
GeneElute	3.2<br>2.2<br>3	3	2.80	3.1	2.6	0.43
innuPREP	0<br>0.1<br>0.1	0.1	0.07	0.1	0.05	0.05
MagMAX	11.6<br>13.7<br>12.4	12.4	12.57	13.05	12	0.87
Nuclisens	0<br>0<br>0	0	0	0	0	0
MagVet	2.4<br>2.3<br>0	2.3	1.57	2.35	1.15	1.11
ID Gene	0<br>0<br>0	0	0	0	0	0

*For detailed DNA isolation kit information see Table S5.

Table S7. Efficiency of MAP DNA isolation from milk.

Kit	Blood and Tissue[a]		MagVet[b]		MagMAX[c]		Nuclisens[d]		BioSprint[e]		EZ1[f]		ZymoResearch[g]	
Input[h]	Output[i]	Efficiency [%]	Output[i]	Efficiency [%]	Output[i]	Efficiency [%]	Output[i]	Efficiency[%]	Output[i]	Efficiency[%]	Output[i]	Efficiency[%]	Output[i]	Efficiency [%]
2.48 × 10^7	7.37 × 10^6	29.7	1.86 × 10^5	0.8	7.99 × 10^5	3.2	1.43 × 10^4	0.1	2.75 × 10^5	1.1	2.01 × 10^4	0.1	4.89 × 10^6	19.7
2.48 × 10^6	2.58 × 10^5	10.4	1.59 × 10^4	0.6	5.66 × 10^4	2.3	7.63 × 10^2	0.0	2.07 × 10^4	0.8	2.02 × 10^3	0.1	2.41 × 10^5	9.7
2.48 × 10^5	1.67 × 10^4	6.7	1.13 ×10^3	0.5	2.77 × 10^3	1.1	7.40 × 10^0	0.0	1.51 × 10^3	0.6	1.86 × 10^2	0.1	2.69 × 10^4	10.8

[a]DNeasy® Blood and tissue kit (QIAGEN, Hilden, Germany); [b]MagVet *Mycobacterium paratuberculosis* Isolation Kit (LSI, Lissieu, France); [c]MagMAX™ Total Nucleic Acid Isolation Kit (Applied Biosystems by Thermo Fisher Scientific, Vilnius, Lithuania); [d]Nuclisens Magnetic Extraction Reagents (Biomérieux, Marcy-l'Étoile, France); [e]BioSprint 96 One-For-All-Vet (QIAGEN, Hilden, Germany); [f]EZ1 DNA Tissue Kit (QIAGEN, Hilden, Germany); [g]ZR Quick-DNA Fecal Soil Microbe Microprep Kit (Zymo Research, Tustin, CA, USA); [h]Input is calculated as the mean number of MAP cells in a 10 µl dose of MAP culture suspension used for artificial contamination; [i]Output is calculated (based on F*57* qPCR) as the mean number of MAP cells per sample recovered after DNA isolation.

Table S8. Comparison of efficiency of MAP DNA isolation from faeces.

Kit		Zymo Research[a]		QIAamp DNA Stool[b]		QIAamp Fast DNA[c] Stool		Power Fecal[d]		Power Soil[e]		NucleoSpin[f]	
Sample type	Input[m]	Output[n]	Efficiency [%]	Output[n]	Efficiency [%]	Output	Efficiency [%]	Output[n]	Efficiency[%]	Output	Efficiency[%]	Output	Efficiency[%]
Faecal sample	5.60 × 10^6	2.87 × 10^6	51.2	1.22 × 10^6	21.8	3.50 × 10^5	6.3	2.30 × 10^6	41.0	2.47 × 10^6	44.2	9.33 × 10^5	16.7
	5.60 × 10^5	2.41 × 10^5	43.0	5.45 × 10^4	9.7	2.92 × 10^4	5.2	1.49 × 10^5	26.5	1.10 × 10^5	19.6	5.68 × 10^4	10.1
	5.60 × 10^4	2.00 × 10^4	35.6	4.19 × 10^3	7.5	5.87 × 10^3	10.5	9.80 × 10^3	17.5	1.17 × 10^4	20.9	3.03 × 10^3	5.4
Faecal pellet	5.60 × 10^6	3.63 × 10^6	64.8	1.48 × 10^6	26.4	1.13 × 10^6	20.1	2.72 × 10^6	48.5	2.38 × 10^6	42.4	2.44 × 10^6	43.5
	5.60 × 10^5	2.95 × 10^5	52.7	6.36 × 10^4	11.3	1.82 × 10^4	3.2	1.26 × 10^5	22.6	1.29 × 10^5	23.0	1.73 × 10^5	30.8

	5.60×10^4	3.01×10^4	53.7	9.95×10^3	17.8	2.98×10^3	5.3	1.05×10^4	18.7	1.13×10^4	20.2	1.41×10^4	25.1

		GeneElute[g]		innuPREP[h]		MagMAX[i]		Nuclisens[j]		MagVet[k]		ID Gene[l]	
Sample type	Input[m]	Output[n]	Efficiency [%]	Output[n]	Efficiency [%]	Output[n]	Efficiency [%]	Output[n]	Efficiency [%]	Output[n]	Efficiency [%]	Output[n]	Efficiency [%]
Faecal sample	5.60×10^6	1.81×10^5	3.2	5.07×10^2	0.0	6.47×10^5	11.6	inhibition	0.0	1.34×10^5	2.4	inhibition	0.0
	5.60×10^5	1.20×10^4	2.2	4.32×10^2	0.1	7.69×10^4	13.7		0.0	1.31×10^4	2.3		0.0
	5.60×10^4	1.70×10^3	3.0	6.73×10^1	0.1	6.95×10^3	12.4		0.0	0.00×10^0	0.0		0.0
Faecal pellet	5.60×10^6	1.33×10^6	23.7	5.69×10^2	0.0	1.06×10^6	19.0	1.10×10^5	2.0	1.71×10^5	3.1	2.22×10^5	4.0
	5.60×10^5	2.05×10^4	3.7	4.00×10^2	0.1	6.97×10^4	12.4	4.15×10^3	0.7	6.94×10^3	1.2	1.00×10^4	1.8
	5.60×10^4	1.26×10^3	2.2	5.24×10^2	0.1	8.48×10^3	15.1	3.64×10^2	0.7	1.36×10^3	2.4	5.87×10^2	1.1

[a]ZR Quick-DNA Fecal Soil Microbe Microprep Kit (Zymo Research, Tustin, CA, USA); [b]QIAamp DNA Stool Mini Kit (QIAGEN, Hilden, Germany); [c]QIAamp Fast DNA Stool Mini Kit (QIAGEN, Hilden, Germany); [d]Power Fecal DNA Kit (QIAGEN, Hilden, Germany); [e]DNeasy Power Soil Kit (QIAGEN, Hilden, Germany); [f]NucleoSpin DNA Stool (Macherey-Nagel, Düren, Germany); [g]Gen Elute Stool DNA Isolation Kit (Sigma-Aldrich, St. Louis, MO, USA); [h]Innu PREP Stool DNA kit (Analytik Jena, Berlin, Germany); [i]MagMAX™ Total Nucleic Acid Isolation Kit (Applied Biosystems by Thermo Fisher Scientific, Vilnius, Lithuania); [j]Nuclisens Magnetic Extraction Reagents (Biomérieux, Marcy-l'Étoile, France); [k]MagVet *Mycobacterium paratuberculosis* Isolation Kit (LSI, Lissieu, France); [l]ID Gene Mag Paratuberculosis Extraction Kit (ID vet Genetics, Grabels, France); [m]Input is calculated as the mean number of MAP cells in a 10 µl dose of MAP culture suspension used for artificial contamination; [n]Output is calculated (based on F*57* qPCR) as the mean number of MAP cells per sample recovered after DNA isolation.

Table S9. Automatic magnetic separation of MAP DNA in milk.

	Mag Universal[a] Extraction Kit		Mag Fast Extraction Kit[b]		MagVet[c]		MagMAX[d]		BioSprint[e]	
Input[f]	Output[g]	Efficiency [%]	Output[g]	Efficiency [%]	Output[g]	Efficiency [%]	Output[g]	Efficiency [%]	Output[g]	Efficiency[%]
1.40×10^7	3.85×10^5	2.8	3.53×10^6	25.3	7.70×10^5	5.5	2.13×10^6	15.3	3.86×10^5	2.8
1.40×10^6	3.80×10^4	2.7	1.55×10^5	11.1	3.37×10^4	2.4	1.07×10^5	7.7	2.12×10^4	1.5
1.40×10^5	1.64×10^3	1.2	9.53×10^3	6.8	1.66×10^3	1.2	6.15×10^3	4.4	5.05×10^2	0.4

[a]ID Gene™ Mag Universal Extraction Kit (ID vet Genetics, Grabels, France); [b]ID Gene™ Mag Fast Extraction Kit (ID vet Genetics, Grabels, France); [c]MagVet *Mycobacterium paratuberculosis* Isolation Kit (LSI, Lissieu, France); [d]MagMAX™ Total Nucleic Acid Isolation Kit (Applied Biosystems by Thermo Fisher Scientific, Vilnius, Lithuania); [e]BioSprint 96 One-For-All-Vet (QIAGEN, Hilden, Germany); [f]Input is calculated as the mean number of MAP cells in a 10 µl dose of MAP culture suspension used for artificial contamination; [g]Output is calculated (based on F*57* qPCR) as the mean number of MAP cells per sample recovered after DNA isolation.

Table 10. Data derived from isolation efficiency of MAP DNA automatic magnetic separation from milk, unit: percentage [%].

Kit	Isolation Efficiency	Median	Mean	Upper Quartile	Lower Quartile	Standard Deviation
MagUniversal[a]	2.8<br>2.7<br>1.2	2.7	2.23	2.75	1.95	0.73
MagFast Extraction[b]	25.3<br>11.1<br>6.8	11.1	14.40	18.2	8.95	7.90
MagVet[c]	5.5<br>2.4<br>1.2	2.4	3.03	3.95	1.8	1.81
MagMAX[d]	15.3<br>7.7<br>4.4	7.7	9.13	11.5	6.05	4.56
BioSprint[e]	2.8<br>1.5<br>0.4	1.5	1.57	2.15	0.95	0.98

[a]ID Gene™ Mag Universal Extraction Kit (ID vet Genetics, Grabels, France); [b]ID Gene™ Mag Fast Extraction Kit (ID vet Genetics, Grabels, France); [c]MagVet *Mycobacterium paratuberculosis* Isolation Kit (LSI, Lissieu, France); [d]MagMAX™ Total Nucleic Acid Isolation Kit (Applied Biosystems by Thermo Fisher Scientific, Vilnius, Lithuania); [e]BioSprint 96 One-For-All-Vet (QIAGEN, Hilden, Germany).

Table S11. Comparison of manual and automatic magnetic separation of MAP DNA in milk.

Isolation type	MagVet[a]			MagMAX[b]		BioSprint[c]	
	Input[d]	Output[e]	Efficiency [%]	Output[e]	Efficiency [%]	Output[e]	Efficiency [%]
Manual	2.48×10^7	1.86×10^5	0.8	7.99×10^5	3.2	2.75×10^5	1.1
	2.48×10^6	1.59×10^4	0.6	5.66×10^4	2.3	2.07×10^4	0.8
	2.48×10^5	1.13×10^3	0.5	2.77×10^3	1.1	1.51×10^3	0.6
Automatic	1.40×10^7	7.70×10^5	5.5	2.13×10^6	15.3	3.86×10^5	2.8
	1.40×10^6	3.37×10^4	2.4	1.07×10^5	7.7	2.12×10^4	1.5
	1.40×10^5	1.66×10^3	1.2	6.15×10^3	4.4	5.05×10^2	0.4

[a]MagVet *Mycobacterium paratuberculosis* Isolation Kit (LSI, Lissieu, France); [b]MagMAX™ Total Nucleic Acid Isolation Kit (Applied Biosystems by Thermo Fisher Scientific, Vilnius, Lithuania); [c]BioSprint 96 One-For-All-Vet (QIAGEN, Hilden, Germany); [d]Input is calculated as the mean number of MAP cells in a 10 µl dose of MAP culture suspension used for artificial contamination; [e]Output is calculated (based on F*57* qPCR) as the mean number of MAP cells per sample recovered after DNA isolation.

Table S12. Comparison of isolation efficiency derived data of MAP DNA manual and automatic magnetic separation from milk, unit: percentage [%].

Isolation Type	Kit	Isolation Efficiency	Median	Mean	Upper Quartile	Lower Quartile	Standard Deviation
Manual	MagVet[a]	0.8<br>0.6<br>0.5	0.6	0.63	0.7	0.55	0.12
	MagMAX[b]	3.2<br>2.3<br>1.1	2.3	2.20	2.75	1.7	0.86
	BioSprint[c]	1.1<br>0.8<br>0.6	0.8	0.83	0.95	0.7	0.21
Automatic	MagVet[a]	5.5<br>2.4<br>1.2	2.4	3.03	3.95	1.8	1.81
	MagMAX[b]	15.3<br>7.7<br>4.4	7.7	9.13	11.5	6.05	4.56
	BioSprint[c]	2.8<br>1.5<br>0.4	1.5	1.57	2.15	0.95	0.98

[a]MagVet *Mycobacterium paratuberculosis* Isolation Kit (LSI, Lissieu, France); [b]MagMAX™ Total Nucleic Acid Isolation Kit (Applied Biosystems by Thermo Fisher Scientific, Vilnius, Lithuania); [c]BioSprint 96 One-For-All-Vet (QIAGEN, Hilden, Germany).

Table S13. Concentration and purity of nucleic acids isolated from milk.

Kit[a]	Theoretical Input [MAP cells/ml]	NA[b] Concentration [ng/µl]	260/280	260/230
Blood and tissue	2.48 × 07	216.4	1.57	0.31
	2.48 × 07	286.1	1.59	0.36
	2.48 × 07	281.3	1.56	0.35
	2.48 × 06	179.0	1.60	0.31
	2.48 × 06	214.2	1.54	0.30
	2.48 × 06	171.3	1.58	0.33
	2.48 × 05	151.6	1.44	0.23
	2.48 × 05	209.6	1.52	0.30
	2.48 × 05	184.4	1.51	0.27
MagVet		11.0	1.21	0.23
		6.4	1.66	0.41
		7.5	1.38	0.29
		1.2	0.69	0.20
		7.4	1.74	0.56
		9.3	1.81	0.83
		12.3	1.77	0.78
		7.9	1.91	0.60
		6.9	1.41	0.34
MagMAX		64.1	2.14	0.10
		93.5	1.75	0.12
		63.3	1.72	0.08
		63.3	1.75	0.10
		93.1	1.97	0.12
		61.9	1.95	0.08
		116.2	2.01	0.19
		58.7	2.04	0.07
		86.4	1.54	0.13
Nuclisens		58.6	1.45	0.17
		29.6	1.36	0.09
		50.1	1.46	0.17
		67.1	1.47	0.22
		80.6	1.48	0.38
		62.3	1.45	0.26

		43.8	1.44	0.14
		40.9	1.49	0.24
		78.8	1.40	0.12

Kit[a]	Theoretical input [MAP cells/ml]	NA[b] concentration [ng/µl]	260/280	260/230
BioSprint		74.8	1.36	0.28
		105.2	0.90	0.17
		56.0	0.87	0.21
		41.3	0.76	0.09
		55.1	0.76	0.08
		93.6	0.86	0.14
		49.4	0.79	0.11
		36.1	0.78	0.12
		44.8	0.76	0.11
EZ1		10.5	1.81	0.13
		10.5	1.54	0.66
		10.1	1.67	0.03
		11.7	1.61	0.26
		8.60	1.40	0.01
		11.7	1.50	0.08
		10.4	1.51	0.07
		8.6	1.54	0.07
		7.0	1.42	0.59
Zymo Research		199.8	1.81	2.03
		181.6	1.82	2.09
		154.7	1.82	2.00
		222.4	1.82	2.20
		182.1	1.82	2.19
		202.9	1.82	2.15
		196.7	1.82	2.19
		193.2	1.81	2.13
		155.9	1.81	2.06

[a]For complete kit information see Supplementary information Table 1; [b]NA = Nucleid Acid

Table S14. Concentration and purity of nucleic acid isolated from faecal pellets.

Kit[a]	Theoretical Input [MAP cells/ml]	NA[b] Concentration [ng/µl]	260/280	260/230
Zymo Research	5.60 × 06	61.9	1.79	1.72
	5.60 × 06	46.9	1.86	1.78
	5.60 × 05	25.4	1.89	1.35
	5.60 × 05	66.6	1.79	1.90
	5.60 × 04	68.3	1.88	1.84
	5.60 × 04	49.2	1.83	1.19
QIAamp DNA Stool		2.0	1.19	0.54
		2.6	1.38	0.57
		2.6	1.63	0.51
		1.9	1.06	0.53
		23.8	1.90	0.39
		3.2	1.36	0.54
QIAamp Fast DNA Stool		4.2	2.08	1.47
		4.6	2.53	2.28
		4.7	2.43	2.79
		4.3	2.39	3.65
		4.7	2.41	1.97
		4.7	2.57	2.38
Power Fecal		11.7	1.78	1.58
		11.5	1.73	1.41
		12.8	1.65	1.43
		15.1	1.84	0.71
		12.5	1.82	1.45
		11.6	1.72	1.63
Power Soil		10.5	1.79	1.1
		12.9	1.80	1.09
		12.1	1.77	1.20
		13.4	1.72	1.06
		12.3	1.81	1.03
		12.3	1.81	1.11
NucleoSpin		42.0	1.55	1.01
		42.4	1.53	0.77
		43.0	1.53	0.92
		42.4	1.54	0.94

		41.1	1.54	0.95
		43.0	1.53	0.91

**Kit**[a]	**Theoretical input** [MAP cells/ml]	**NA**[b] **concentration** [ng/µl]	**260/280**	**260/230**
GenElute	5.60 × 06	65.7	1.44	0.51
	5.60 × 06	66.3	1.50	0.59
	5.60 × 05	59.4	1.51	0.23
	5.60 × 05	63.1	1.52	0.52
	5.60 × 04	62.0	1.52	0.55
	5.60 × 04	61.5	1.50	0.37
innuPREP		39.3	1.57	0.64
		61.2	1.5	0.48
		31.9	1.61	0.51
		33.4	1.62	0.64
		30.4	1.63	0.60
		23.2	1.62	0.65
MagMAX		11.4	2.23	0.03
		10.8	2.21	0.09
		10.9	2.34	0.14
		9.8	2.51	0.26
		11.0	2.22	0.17
		13.9	2.16	0.28
Nuclisens		83.0	1.34	0.12
		100.2	1.27	0.32
		121.0	1.29	0.16
		97.9	1.33	0.14
		110.0	1.29	0.17
		112.1	1.28	0.19
MagVet		29.9	1.31	0.50
		34.2	1.31	0.52
		34.4	1.34	0.53
		29.4	1.31	0.52
		34.2	1.35	0.54
		36.6	1.33	0.52
ID Gene		36.3	1.37	0.55

	37.6	1.38	0.54
	41.3	1.39	0.55
	33.7	1.34	0.53
	50.0	1.39	0.55
	32.8	1.34	0.52

[a]For complete kit information see Supplementary information Table 2; [b]NA = Nucleid Acid.

Table S15. Concentration and purity of nucleic acid automatically isolated from milk.

Kit[a]	Theoretical Input [MAP cells/ml]	NA[b] Concentration [ng/µl]	260/280	260/230
Mag Universal Extraction Kit	1.40 × 07	11.9	1.61	0.83
	1.40 × 07	11.4	1.63	1.24
	1.40 × 07	13.2	1.65	1.27
	1.40 × 06	13.8	1.80	1.38
	1.40 × 06	16.8	1.74	1.90
	1.40 × 06	23.0	1.81	1.68
	1.40 × 05	21.9	1.77	1.83
	1.40 × 05	11.6	1.76	1.65
	1.40 × 05	18.2	1.70	1.20
Mag Fast Extraction kit		27.1	1.50	0.76
		25.6	1.50	0.85
		29.5	1.57	0.99
		30.4	1.54	0.92
		27.0	1.52	0.85
		25.6	1.50	0.77
		27.5	1.57	0.83
		30.0	1.65	0.93
		25.0	1.50	0.88
MagVet		9.7	1.54	0.90
		8.5	1.55	0.91
		10.9	1.55	1.00
		10.7	1.60	0.93
		7.7	1.76	0.92
		11.4	1.53	0.85
		9.2	1.56	0.78
		10.6	1.50	0.77
		8.6	1.60	0.71
MagMAX		34.3	2.16	1.33
		29.4	2.16	1.51
		42.5	2.07	1.10
		41.9	2.06	1.28
		35.1	2.04	1.14
		35.6	2.12	1.19
		30.3	2.08	1.37
		29.0	2.28	1.79
		29.1	2.12	1.44

BioSprint	35.9	1.76	0.74
	32.9	1.68	0.74
	43.3	1.70	0.56
	34.1	1.76	0.56
	42.9	1.76	0.69
	37.2	1.86	0.78
	23.5	2.12	1.35
	32.2	1.85	0.96
	12.8	2.21	1.62

[a]For complete kit information see Supplementary information Table 3; [b]NA = Nucleid Acid.

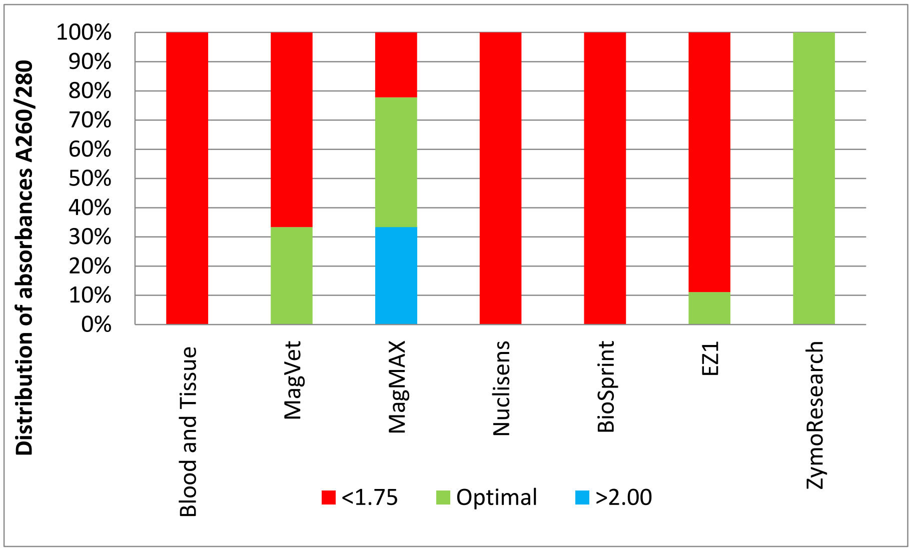

Figure S1. Distribution of DNA purity values of selected isolation kits for MAP DNA isolation from milk.

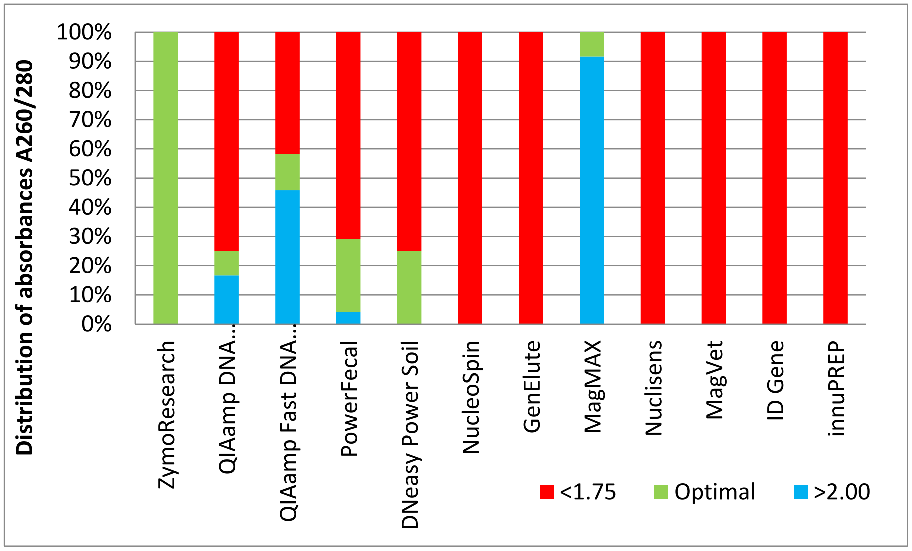

Figure S2. Distribution of DNA purity values of selected isolation kits for MAP DNA isolation from faeces.

© 2020 by the authors. Licensee MDPI, Basel, Switzerland. This article is an open access article distributed under the terms and conditions of the Creative Commons Attribution (CC BY) license (http://creativecommons.org/licenses/by/4.0/).
